# HIF-1-mediated macrophage metabolic reprogramming promotes AKI to CKD transition

**DOI:** 10.7150/ijbs.111238

**Published:** 2025-09-22

**Authors:** Hong Ding, Yan Zhou, Ren-He Zhu, Shu Yu, Ai-Qing Zhang, Hong Liu, Jia-Ling Ji, Zuo-Lin Li

**Affiliations:** 1 Institute of Nephrology, Zhong Da Hospital, Southeast University School of Medicine, Nanjing, Jiangsu, P.R. China.; 2 Institute of Nephrology, People's Hospital of Yangzhong city, Zhenjiang, Jiangsu, P.R. China.; 3 Institute of Nephrology, Affiliated Hospital of Jiangsu University, Zhenjiang, Jiangsu, P.R. China.; 4 Department of Pediatrics, The Fourth Affiliated Hospital of Nanjing Medical University, Nanjing, Jiangsu, P.R. China.

**Keywords:** macrophage, metabolic reprogramming, acute kidney injury, chronic kidney disease, hypoxia-inducible factor-1, NF-κB

## Abstract

Macrophage is educated by the tubule epithelial cell with maladaptive repair during the renal maladaptive repair, which is one of the most important characteristic features in acute kidney injury (AKI) to chronic kidney disease (CKD) transition. However, the underlying mechanism of orchestrating characterization of macrophage in renal maladaptive repair remains largely unclear. Accordingly, we found that pro-inflammatory macrophage educated by tubule epithelial cell with maladaptive repair was the primary contributor to the renal maladaptive repair in AKI to CKD transition, because macrophages depletion significantly attenuated tubulointerstitial fibrosis. Meanwhile, we found that glycolysis was essential for maintaining pro-inflammatory macrophage phenotype. Further, we demonstrated that HIF-1α played a crucial role in macrophage glycolysis as myeloid *HIF-1α* knockout alleviated tubulointerstitial fibrosis and AKI to CKD transition *in vivo*. Mechanistically, NF-κB directly binds to the HIF-1α promoter, boosting its transcription and significantly contributing to tubulointerstitial fibrosis in the AKI to CKD transition. Blockage of NF-κB ameliorated the CKD progression following AKI *in vivo*. Taken together, our studies provide a novel paradigm in which pro-inflammatory macrophage orchestrates renal maladaptive repair, contributing to the AKI to CKD transition. Blockade of NF-κB-HIF-1 signaling-mediated macrophage metabolic reprogramming may provide attractive strategy for pharmacologic therapy of the AKI to CKD transition.

## Introduction

Acute kidney injury (AKI), a clinical syndrome characterized by a rapid decline or loss of renal function, is associated with substantial morbidity and mortality 1-2. More importantly, AKI significantly increases the risk for the development of chronic kidney disease (CKD) 2-3. Histologically, renal maladaptive repair following AKI is recognized as a predictive factor for progression to CKD 4-5. However, the mechanism of renal maladaptive repair remains obscure. Elucidating the exact mechanism of maladaptive repair not only deepens the understanding of the underlying pathophysiology of AKI to CKD transition, but also provides a promising therapeutic strategy for delaying the progression of AKI to CKD.

Packed with mitochondria and dependent on oxidative phosphorylation, the tubule epithelial cell (TEC) is particularly vulnerable to injury (ischemic, hypoxic, oxidative, and metabolic) 6-7. Indeed, the TEC represents the central cell type affected by AKI 7-9. Accumulating evidence indicated that due to its notable regenerative capacity, the response of TEC determines the outcomes of AKI. Accordingly, the adaptive repair following AKI facilitates renal recovery. Conversely, when injury is severe or persistent, it allows the TECs to undergo maladaptive repair and phenotypic transformation, thus, contributing to the development of CKD 10-11. Hence, TEC with maladaptive repair is a key instigator of renal maladaptive repair. However, the molecular mechanism of maladaptive TEC-mediated AKI to CKD transition requires elucidation.

Compelling evidence suggests that maladaptive repair of TEC created a unique profibrotic microenvironment through cell-cell crosstalk 12. Especially, infiltrated immune cell is one of the most important contributors. For instance, Jia *et al.* 13 found that in the setting of ischemia-reperfusion injury (I/RI), injured tubular cells recruited immune cells infiltration by secreting specific inflammatory factors, contributing to tubulointerstitial fibrosis. Meanwhile, our group also demonstrated that exosome mediated the cross-talk between injured TEC and macrophage, leading to tubulointerstitial inflammation 14. Hence, as a sentinel cell of the innate immune system, macrophage is a crucial contributor to renal maladaptive repair 15-16. Interestingly, the activation and functional state of the macrophage depends on the stage of tissue injury and repair, reflecting a dynamic and diverse spectrum of macrophage phenotypes 17-18. Although previous studies have suggested that macrophage played a pivotal role during the AKI to CKD transition, the underlying mechanism of orchestrating characterization of macrophage remains poorly understood.

Here, using the I/RI-induced AKI to CKD transition *in vivo* and the tubule-macrophage co-culture* in vitro* model, the underlying mechanism of renal maladaptive repair following AKI was explored. Interestingly, we found that pro-inflammatory macrophage, which was educated by TEC with maladaptive repair, plays a critical role in the AKI to CKD transition. Mechanistically, NF-κB/hypoxia-inducible factor-1 (HIF-1) signaling pathway-mediated metabolic reprogramming was the exact molecular mechanism for shaping this phenotype, contributing to the tubulointerstitial fibrosis and AKI to CKD transition. Therefore, our findings not only represent novel insights into the pathogenesis of the renal maladaptive repair, but also provide a promising therapeutic strategy for delaying the progression of CKD.

## Methods and Materials

### Animals

Male C57BL/6J mice (6~8 weeks old, Vital River Laboratory Animal Technology Co., Ltd., Beijing, China) were housed in a pathogen-free animal care facility in a temperature-controlled room (20 ± 1°C, relative humidity 45%~65%) and had free access to chow and water under a 12 hours light/dark cycle. Mouse bilateral I/RI model was established as previously described 19. In brief, the mice were anesthetized with isoflurane. Bilateral renal pedicles were clamped with non-traumatic artery clamps for 28 or 35 minutes, and then reperfusion was achieved by removing the clamps to induce recovery AKI or AKI to CKD transition model, respectively. During the ischemic period, the mouse body temperature was monitored and kept at 36.5~37 °C using a temperature-controlled heating system. Sham control mice underwent the same surgical procedures without clamping of renal pedicles. For a longitudinal analysis to determine proper models, Mice were killed under general anesthesia either on days 7, 14, 21, and 28 after I/RI for a longitudinal analysis (5 mice per group) , or on day 21 after I/RI for following research (6 mice per group), and their serum and kidneys were harvested. For unilateral ureteral obstruction (UUO, 6 mice per group), the right ureter of each mouse was ligated just below the renal pelvis through a back incision. Sham mice underwent the same surgical procedures, except that the ureter was not ligated. Mice were killed on day 10 after surgery under general anesthesia, and their kidneys were harvested.

Myeloid cell-specific* HIF-1α* depleted mice on the C57BL/6J background (HIF-1α^flox/flox^ LysM-Cre^+/-^: *HIF-1α KO*) and control mice (HIF-1α^flox/flox^ LysM-Cre^-/-^: *WT*) were obtained by crossing HIF-1α^flox/flox^ mice with mice expressing Cre recombinase under the control by the Lysozyme M promoter (Cyagen Biosciences Inc., Guangzhou, China).

To deplete macrophage *in vivo*, clodronate liposomes (200 µL) or vehicle (phosphate buffered saline, PBS) was administrated via the tail vein injection beginning on day 10 after I/R surgery or on day 5 after UUO surgery, and then 100 µL clodronate liposomes or vehicle was injected via the tail vein every other day thereafter. To inhibit NF-κB, beginning on day 10 after I/RI or on day 5 after UUO surgery, mice were subjected to daily intraperitoneal injections of BAY11-7082 (Selleck Chemicals LLC, Houston, TX, USA) at 5 mg/kg body weight or vehicle (PBS) for 10 consecutive days. All mice were euthanized on day 21 after I/RI or on day 10 after UUO. Serum and kidney tissues were collected and processed for further analysis.

All procedures for care and use of animals were reviewed and approved by the Ethics Review Committee for Animal Experimentation of Southeast University.

### Cell culture

The immortalized mouse tubular epithelial cells line (mTEC, a gift from Dr. Jeffery B. Kopp, National Institutes of Health) was cultured in DMEM/F12 (Gibco, New York, USA) supplemented with 10% fetal bovine serum (FBS) and 1% penicillin-streptomycin (Gibco). Then, the cells were incubated in a humidified incubator with 5% CO_2_ at 37 °C. Bone-marrow derived macrophage (BMDM) were harvested from femurs of male C57BL/6J mice or Myeloid cell-specific *HIF-1α* knockout C57BL/6J mice and differentiated into macrophages using RPMI-1640 medium supplemented with 10% FBS, 100 IU/mL penicillin, and 100 ng/mL mouse recombined M-CSF (MedChemEpress, Shanghai, China) for 4-7 days at 37 °C in a 5 % CO_2_ incubator. After treatment of 24 hours, the cells were collected and molecular biological experiments were further performed.

### Co-culture experiments and treatment

To mimic the crosstalk between TECs and macrophages, Transwell Permeable Support systems (Corning, USA) for 12-well plates with a 0.4 µm pore-size filter were used as the manufacturer's protocols. The TECs were plated in upper chamber, while recipient macrophages were then seeded in 12-well plates. To mimic AKI to CKD transition condition *in vivo*, the co-cultured experiments were conducted in the presence of hypoxia (1% O_2_) 12 hours—reoxygenation 2 hours—hypoxia 12 hours—reoxygenation 10 hours (Long hypoxia), then, the cells were harvested. As for the control condition, the co-cultured experiments were conducted in the presence of hypoxia (1% O_2_) 12 hours—reoxygenation 24 hours (Short hypoxia). For glycolysis or NF-κB inhibition, the BMDM was treated with the specific glycolysis inhibitor 2-deoxy-d-glucose (2-DG, 10 mM, Selleck Chemicals LLC, Houston, TX, USA) or the specific NF-κB inhibitor BAY11-7082 (10 μM, Selleck Chemicals LLC) at appropriate time points.

### Kidney histology

For histology analysis, kidneys were fixed with 4% formalin, embedded in paraffin, and sectioned to 4-μm thickness. Periodic acid-Schiff staining and Masson's trichrome staining was performed.

### Lactate and pyruvate measurement and HK II activity assay

The renal cortex samples or cells were used for detection. The lactate (S0208S, Beyotime Biotechnology, Shanghai, China), pyruvate (S0299S, Beyotime Biotechnology, Shanghai, China) levels and the HK II activity (ab136957, Abcam, USA) were measured using the commercial kit according to the manufacturer's instructions.

### Western blotting

Kidney cortex sample, and the cells were lysed in RIPA lysis buffer (Servicebio, Wuhan, China), and protein concentration was measured using a BCA assay (Beyotime, Shanghai, China). Equal concentrations and volumes of protein were loaded using sodium dodecyl sulfate-polyacrylamide gel electrophoresis and transferred to polyvinylidene fluoride membranes (Millipore, Massachusetts, USA). Then, following primary antibodies against α-SMA (ab5694, Abcam, USA), collagen-1 (ab34710, Abcam, USA), fibronectin (ab2413, Abcam), β-actin (AB2001, Abways, Shanghai, China), Hexokinase II (HK II, ab209847, Abcam, USA), glucose transporter type 1 (GLUT1, ab115730, Abcam), iNOS (18985-1-AP, ProteinTech, Wuhan, China), Mincle (A20156, ABclonal, Wuhan, China), Phospho-p65 (#3033, CST), p65 (#8242, CST) and HIF-1α (ab2185, Abcam) were used. Finally, after incubation with horseradish peroxidase-conjugated anti-mouse or anti-rabbit IgG (CST) for 2 hours at room temperature, the blots were detected with the chemiluminescence advanced system (GE Healthcare, USA).

### mRNA isolation and real-time PCR

Total RNA from the kidney tissue and BMDM cells were extracted using TRIzol reagent (Vazyme, Nanjing, China), and then, RNA concentration was measured using a NanoDrop 2000 spectrophotometer (NanoDrop Technologies, Wilmington, DE, USA). Next, mRNA was reverse transcribed using a PrimeScript RT reagent kit (Takara) according to the manufacturer's instructions. Finally, relative mRNA expression was detected using a QuantStudio 3 real time PCR System following the manufacturer's instructions (Thermo fisher). The data were normalized to the expression of β-actin mRNA. The gene primer sequences were shown in Table 1.

### Chromatin Immunoprecipitation

Chromatin immunoprecipitation (ChIP) assay was conducted using the Simple ChIP Plus Enzymatic Chromatin IP Kit (Magnetic Beads, #9003, Cell Signaling Technology) according to the manufacturer's instructions. In brief, immunoprecipitation was performed with the antibody against NF-κB p65 (#6956, CST) and irrelevant IgG was used as a control. Precipitated DNAs were identified by real-time PCR using the following specific primers of the promoter region of HIF-1α: forward 5'-GAACAGAGAGCCCAGCAGAG-3' and reverse 5'-TGTGCACTGAGGAGCTGAGG-3'.

### Immunofluorescence and immunohistochemistry

The kidneys were fixed with 4% formalin, embedded in paraffin, and sectioned to 4-μm thickness. Then, immunofluorescence and immunohistochemistry were performed using established methods 20. Here, the following primary antibodies were used: anti-HIF-1α (ab2185, Abcam), anti-iNOS (18985-1-AP, ProteinTech, Wuhan, China), anti-CD68 (ab955, Abcam), and anti-F4/80 (ab6640, Abcam, Cambridge, MA). The kidney tissues were subsequently analyzed with a streptavidin peroxidase detection system (Maixin Technology Co., Ltd., Fuzhou, China) according to the manufacturer's protocols. Images were obtained by laser scanning confocal microscopy (FV-1000, Olympus, Tokyo, Japan).

### Statistical analyses

The data were presented as means ± standard deviations. Two groups of data were analyzed using the *student's t-test*, and the data from more than two groups were compared using *a one-way analysis of variance* followed by *Bonferroni correction*. Here, the GraphPad Prism 8 (GraphPad Software Inc., San Diego, CA, USA) was used. A two-sided p value of < 0.05 was considered significant.

## Results

### Increased macrophage infiltration was associated with I/RI-induced AKI to CKD transition

**Figure 1:**
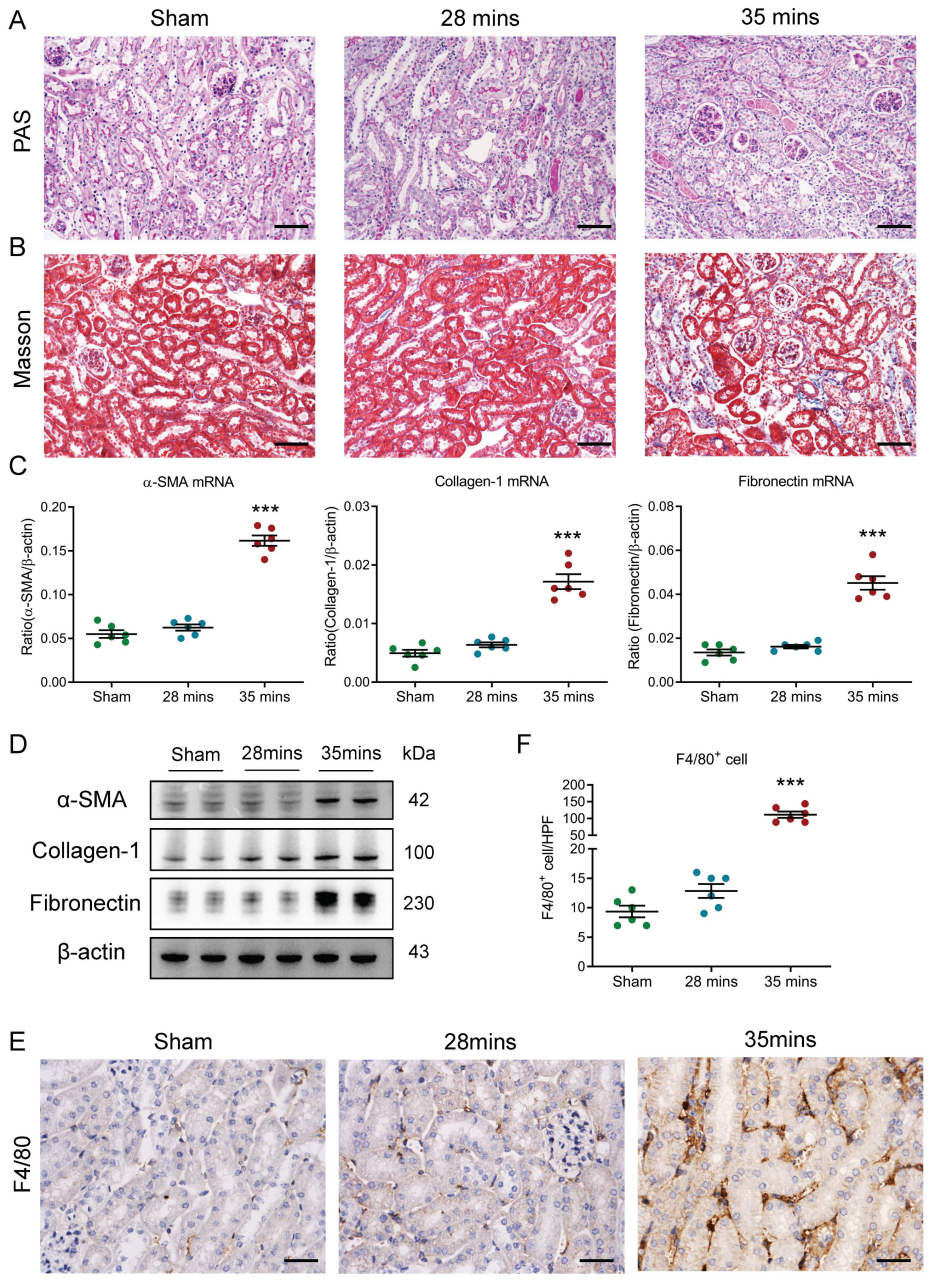
** Increased macrophage infiltration was found in AKI to CKD transition.** (A) Histological changes (Periodic Acid-Schiff staining). Scale bars, 100 μm. (B) Masson's trichrome staining of kidney with I/RI. Scale bar, 100 μm. (C) Real-time PCR analysis of the mRNA expression levels of α-SMA, collagen-1 and fibronectin in kidney (n=6). (D) Western blotting analysis of α-SMA, collagen-1 and fibronectin of kidney (n=6). (E) F4/80 immunohistochemical staining of kidney from mice with I/RI. Scale bars, 50 μm. (F) Quantitation of F4/80+ macrophages infiltrating the kidney of I/RI (n=6). All data are represented as means ± SD. Compared to Sham group, ***P <0.001.

Firstly, I/RI-induced AKI to CKD transition model was established and confirmed. We observed that the levels of serum creatinine (SCr) were similar to the baseline on day 21 in mice with 28 minutes I/RI (recovery AKI); in contrast, the SCr levels were significantly increased on day 21 in mice with 35 minutes I/RI (AKI to CKD transition, supplementary Fig. 1A), suggesting the progression of CKD following AKI. Consistent with this result, as shown in Fig. 1A and B, necrosis and detachment of tubular epithelial cells, cellular debris accumulation, and casts formation and renal fibrosis were observed on day 21 after 35 minutes I/RI, whereas morphologic lesions of tubules recovered in mice with 28 minutes I/RI. Furthermore, the expression of the fibrotic markers α-SMA, collagen-1 and fibronectin at the transcriptional level were markedly increased in kidneys with 35 minutes I/RI (Fig. 1C). Similar results at the protein level were observed in kidney cortex (Fig. 1D and supplementary Fig. 1B). Moreover, a longitudinal analysis of fibrosis markers at multiple time points post-I/RI showed a similar trajectory (Supplementary Fig. 1C-1D). Next, the macrophage infiltration was assessed in kidneys with I/RI. As shown by immunohistochemistry with antibodies against F4/80 in Fig. 1E and 1F, compared with kidney with 28 minutes I/RI, kidney with 35 minutes I/RI exhibited significantly increased macrophage infiltration. Collectively, these results indicate that the progression of CKD after AKI might be associated with increased macrophage infiltration.

### Increased macrophage infiltration was found in the UUO-induced AKI to CKD transition

**Figure 2:**
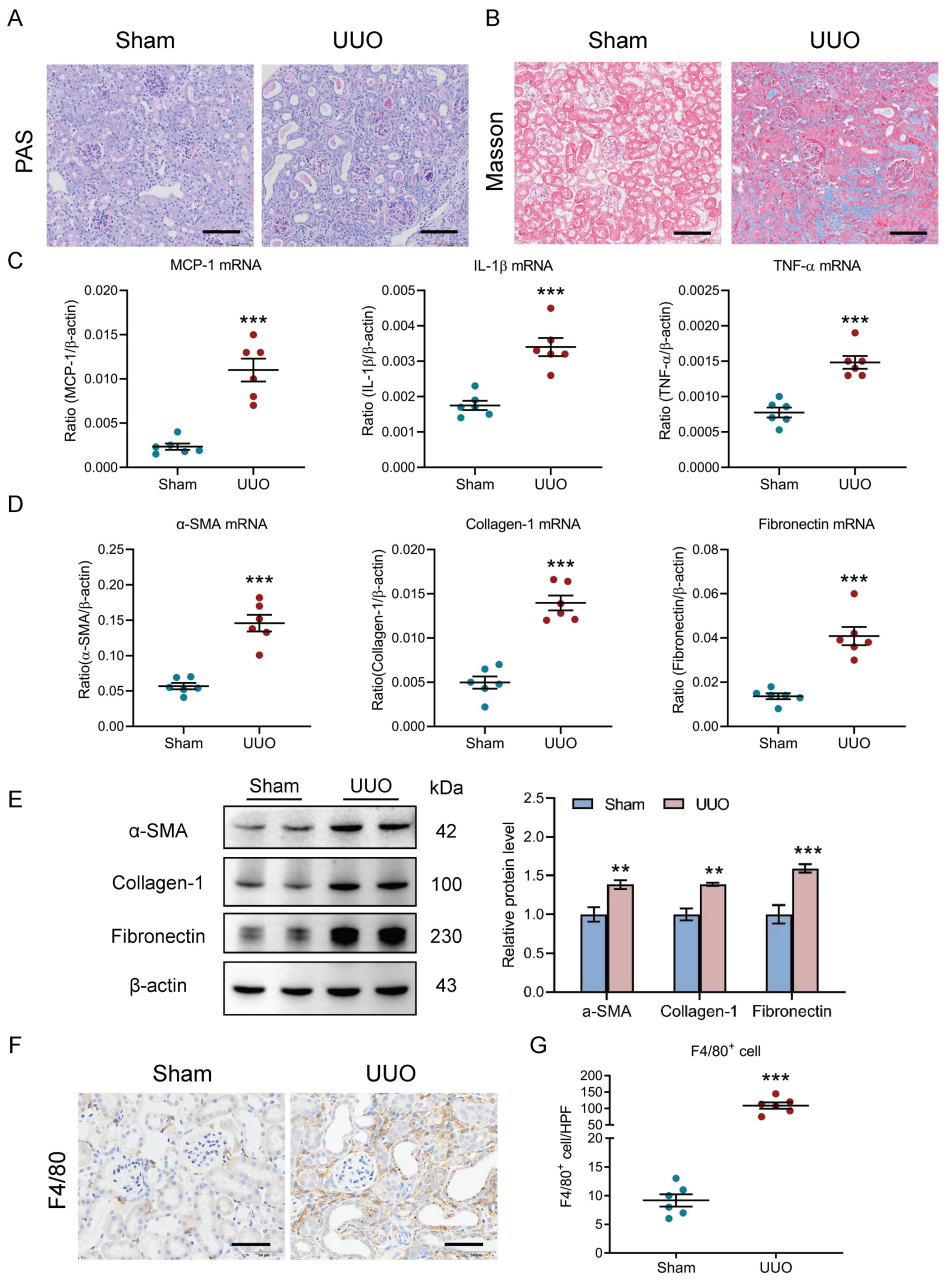
** Increased macrophage infiltration was observed in the UUO-induced AKI to CKD transition.** (A) Histological changes (Periodic Acid-Schiff staining). Scale bars, 100 μm. (B) Masson's trichrome staining of kidney with UUO. Scale bar, 100 μm. (C) Real-time PCR analysis of the mRNA expression levels of MCP-1, IL-1β and TNF-α in kidney (n=6). (D) Real-time PCR analysis of the mRNA expression levels of α-SMA, collagen-1 and fibronectin in kidney (n=6). (E) Western blotting analysis and densitometric analysis of α-SMA, collagen-1 and fibronectin of kidney (n=6). (F) F4/80 immunohistochemical staining of kidney from mice with I/RI. Scale bars, 50 μm. (G) Quantitation of F4/80+ macrophages infiltrating the kidney from mice with UUO (n=6). All data are represented as means ± SD. Compared to Sham group, **P <0.01, ***P <0.001.

The relationship between renal outcomes and macrophage infiltration was further explored in UUO, a nonreversible AKI to CKD transition model. As in the I/R model, TECs injury and protein casts and renal fibrosis were found in kidneys with UUO (Fig. 2A-2B). In parallel, there was significant induction of renal inflammatory cytokines (MCP-1, IL-1β, and TNF-α, Fig. 2C) and fibrotic markers (α-SMA, collagen-1, and fibronectin, Fig. 2D) expression at the transcriptional level. Similar results on fibrotic markers at the protein level were observed in kidney cortex (Fig. 2E). Concomitantly, immunohistochemistry analysis revealed that a remarkable increased macrophages were observed in the kidney with UUO on day 10 (Fig. 2F). Thus, as in I/R, these results highlight a link between the renal outcomes and increased macrophage infiltration in UUO-induced AKI to CKD transition.

### Pro-inflammatory macrophages are observed during AKI to CKD transition

**Figure 3:**
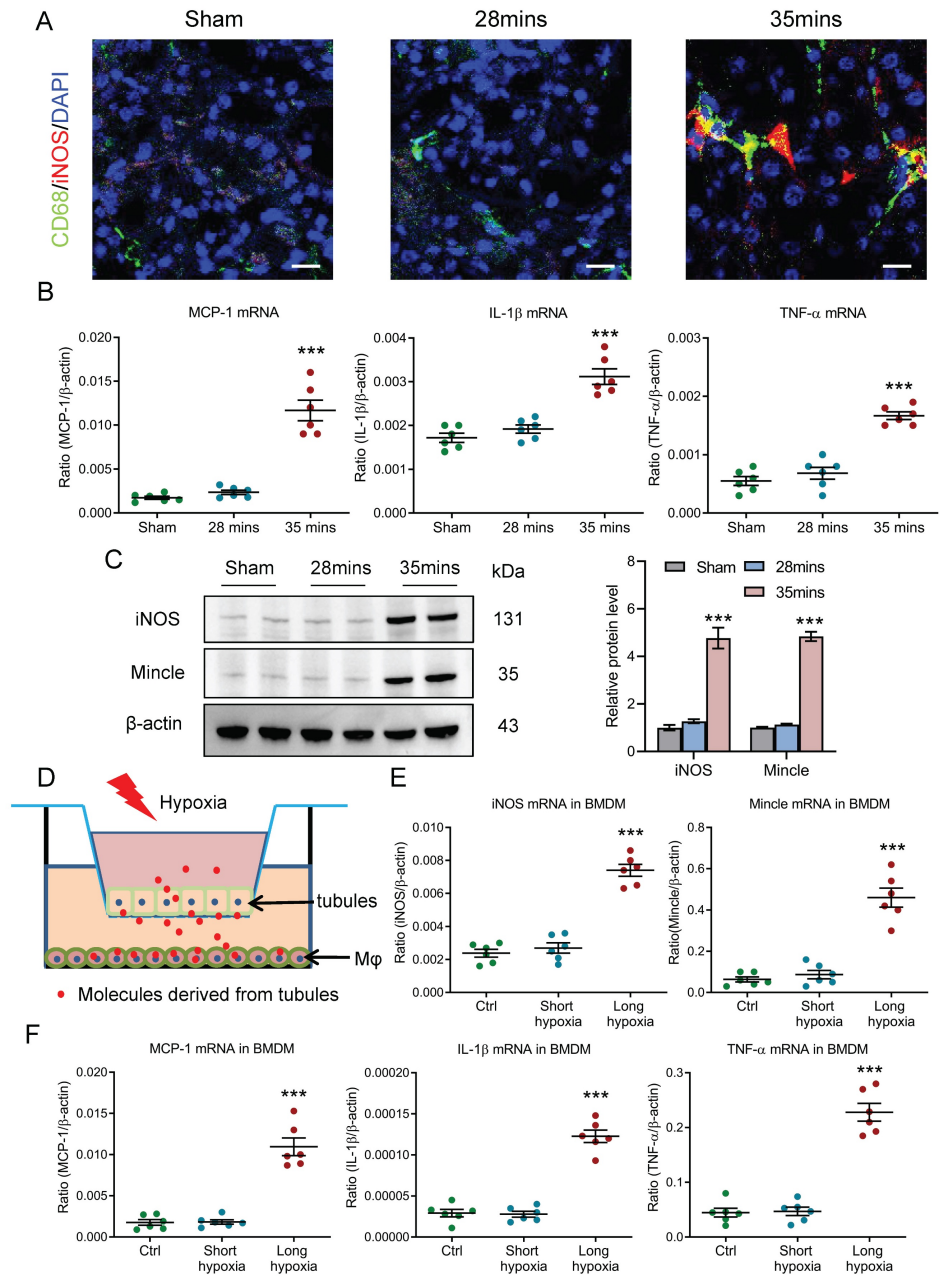
**Macrophages with pro-inflammatory phenotype are observed in AKI to CKD transition**. (A) CD68 (Green) and iNOS (Red) immunohistochemical staining in kidney of I/RI. Nuclei were revealed using 4',6-diamidino-2-phenylindole (DAPI) staining. Scale bars, 20 μm. (B) Real-time PCR analysis of the mRNA expression levels of MCP-1, IL-1β, and TNF-α in kidney with I/RI (n=6). (C) Western blotting analysis and densitometric analysis of iNOS and Mincle in kidney (n=6). (D) Schematic illustration of the mTECs and macrophage co-culture *in vitro* experiment. (E) Real-time PCR analysis of the mRNA expression levels of iNOS and Mincle in BMDM with co-culture under short or long hypoxia (n=6). (F) Real-time PCR analysis of the mRNA expression levels of MCP-1, IL-1β, and TNF-α in BMDM with co-culture under short or long hypoxia (n=6). All data are represented as means ± SD. Compared to Sham or short hypoxia group, ***P <0.001.

Given the diverse spectrum of macrophage phenotypes, we then investigated the potential macrophage phenotype in kidney with AKI to CKD transition. Interestingly, we found that the macrophage with pro-inflammatory phenotypes was markedly increased in the AKI to CKD transition group, as evidenced by the immunostaining of specific pro-inflammatory phenotype marker iNOS (Fig. 3A). Meanwhile, as shown in Fig. 3B, inflammatory factors such as MCP-1, IL-1β and TNF-α at the transcriptional level were also significantly increased in mice with 35 minutes I/RI on day 21. A longitudinal analysis of inflammatory factors at multiple time points post-I/RI showed a similar trajectory (Supplementary Fig. 2A). Interestingly, a similar expression pattern of iNOS and Mincle (specific pro-inflammatory macrophage phenotype marker) was also observed in the I/RI- and UUO-induced AKI to CKD transition models, detected by western blotting (Fig. 3C and supplementary Fig. 2B), suggesting pro-inflammatory macrophages are found in AKI to CKD transition. Furthermore, the macrophage phenotype was then explored by an* in vitro* study. As shown in Fig. 3D, the mTECs and macrophage were co-cultured to mimic *in vivo* conditions. Interestingly, we found that pro-inflammatory phenotype marker iNOS and Mincle (Fig. 3E) and inflammatory factors such as MCP-1, IL-1β and TNF-α at the transcriptional level were markedly increased in BMDM from co-culture system with long-hypoxia administration (Fig. 3F). Together, these results demonstrate that macrophages with pro-inflammatory phenotype are involved in AKI to CKD transition.

### Depletion of macrophages attenuates tubulointerstitial fibrosis in AKI to CKD transition

**Figure 4:**
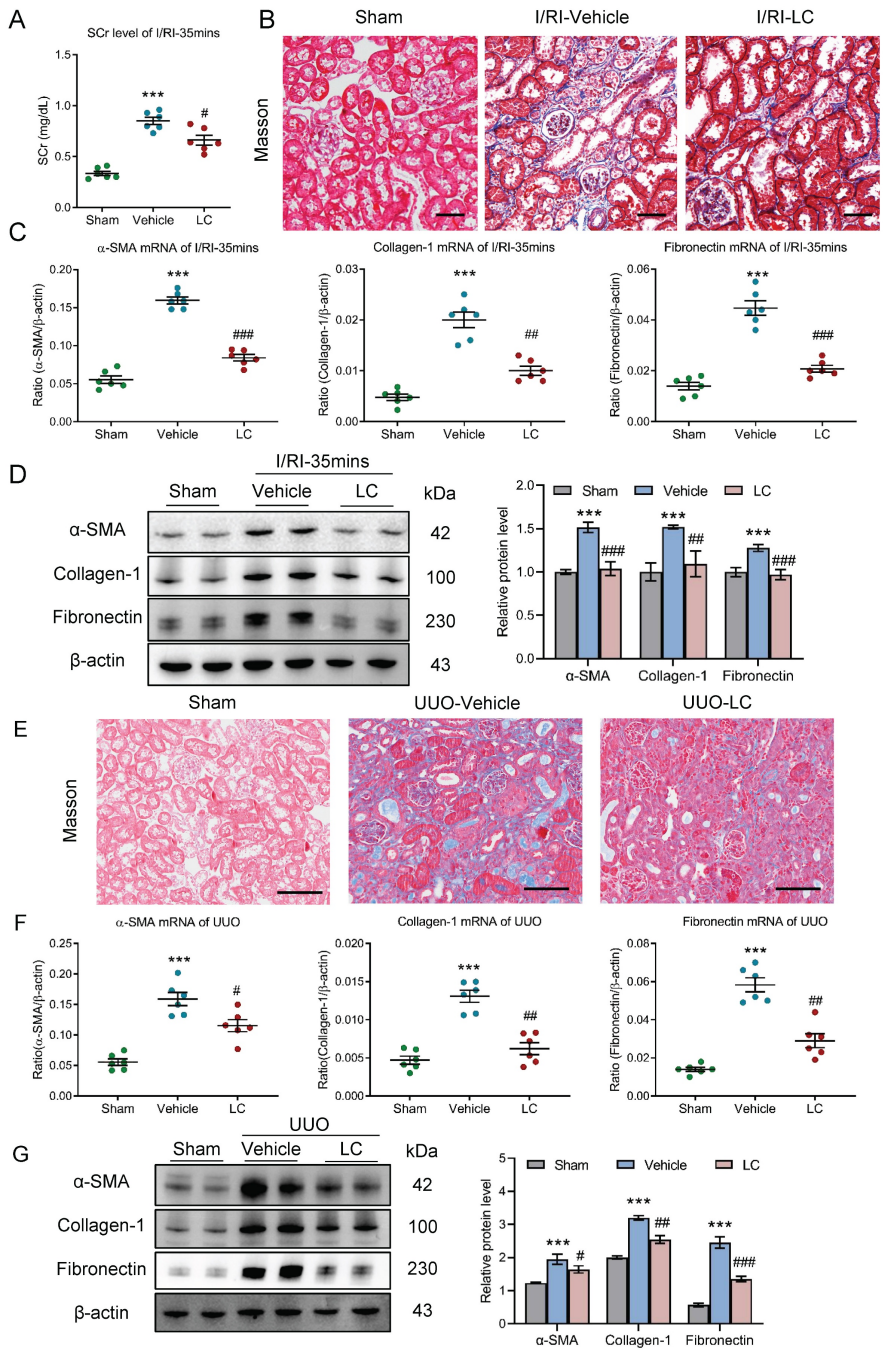
** Depletion of macrophages attenuates tubulointerstitial fibrosis in AKI to CKD transition.** (A) The SCr levels in mice with I/RI (n=6). (B) Histological changes (Masson's trichrome staining) of kidney from I/RI mice with Clodronate liposomes administration. Scale bars, 50 μm. (C) Real-time PCR analysis of the mRNA expression levels of α-SMA, collagen-1 and fibronectin in kidney (n=6). (D) Western blotting analysis and densitometric analysis of α-SMA, collagen-1 and fibronectin of kidney (n=6). (E) Histological changes (Masson's trichrome staining) of kidney from UUO mice with Clodronate liposomes administration. Scale bars, 100 μm. (F) Real-time PCR analysis of the mRNA expression levels of α-SMA, collagen-1 and fibronectin in kidney (n=6). (G) Western blotting analysis and densitometric analysis of α-SMA, collagen-1 and fibronectin of kidney from UUO mice with Clodronate liposomes administration (n=6). All data above are represented as means ± SD. Compared to Sham group, ***P < 0.001; Compared to Vehicle group, #*P* <0.05, ##*P* <0.01, ###*P* <0.001.

We then investigated the potential contribution of macrophages to progression of CKD following AKI using clodronate liposomes, which is known to induce macrophage depletion *in vivo*. Strikingly, we found that the SCr level on day 21 after 35 minutes I/RI was significantly decreased in the mice that received the clodronate liposomes treatment (Fig. 4A). Similarly, Masson staining also demonstrated ameliorated fibrotic lesions (Fig. 4B). Moreover, depletion of macrophages attenuates the increased expression of α-SMA, collagen-1, and fibronectin at the mRNA and protein level in renal cortical tissues, as demonstrated by PCR and Western blotting (Fig. 4C-4D).

Then, the outcomes of depleting macrophages using clodronate liposomes were also validated in UUO-induced AKI to CKD transition model. As expected, ameliorated fibrotic lesions were observed (Fig. 4E). Concomitantly, the expression of α-SMA, collagen-1, and fibronectin at the mRNA level was markedly decreased in the obstructed kidney on day 10 (Fig. 4F), a pattern that was recapitulated by Western blotting (Fig.4G). Collectively, these data indicate that depletion of macrophages efficiently attenuated tubulointerstitial fibrosis and protected the kidney against CKD progression after AKI.

### Glycolysis is essential for maintaining pro-inflammatory macrophage phenotype

**Figure 5:**
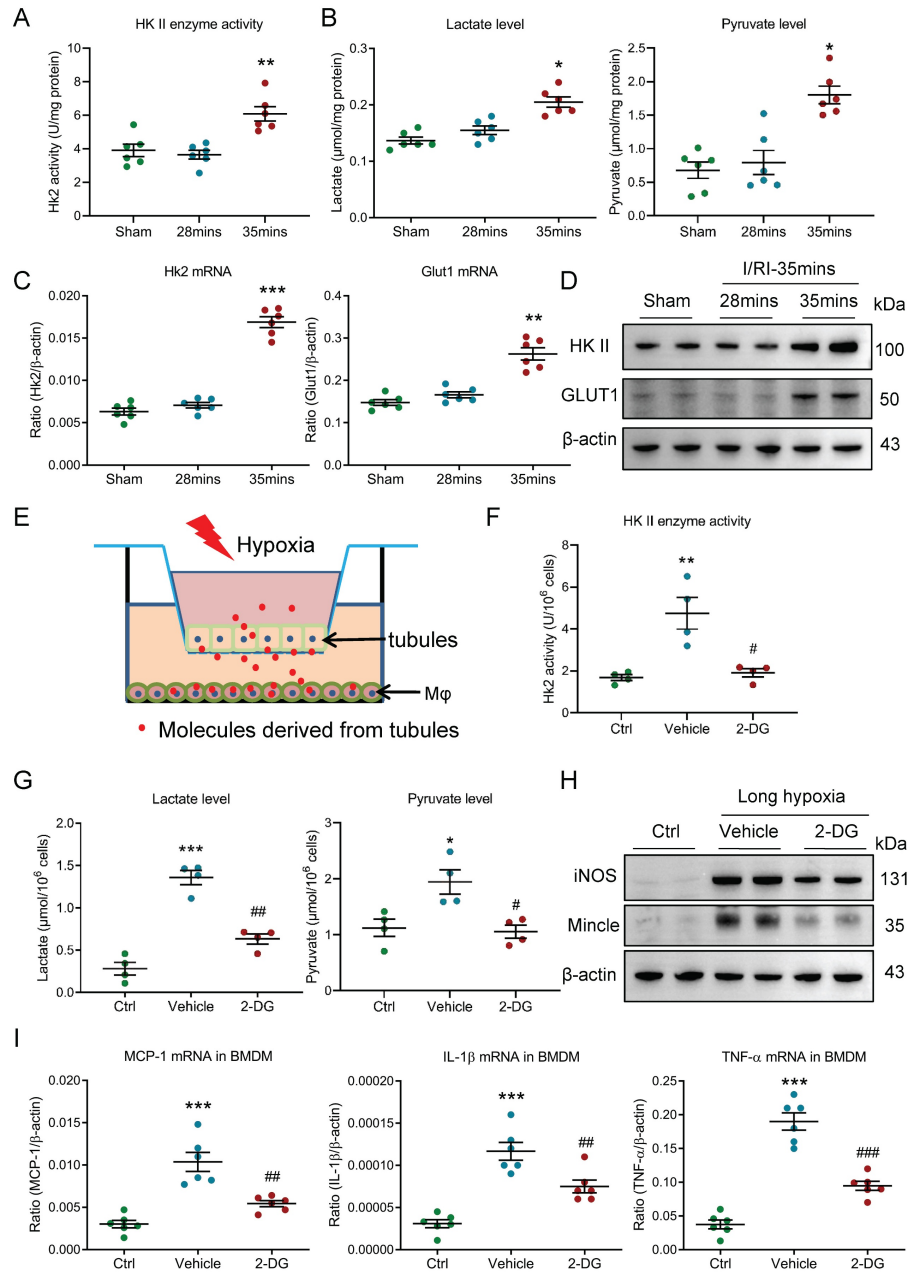
** Glycolysis is essential for maintaining pro-inflammatory macrophage phenotype.** (A) The HK II enzyme activity in kidney with I/RI (n=6). (B) The lactate and pyruvate levels in kidney with I/RI (n=6). (C) Real-time PCR analysis of the mRNA expression level of HK II and GLUT1 in kidney from mice with I/RI (n=6). (D) Western blotting analysis and densitometric analysis of HK II and GLUT1 in kidney (n=6). (E) Schematic illustration of the mTECs and macrophage co-culture* in vitro* experiment. (F) The HK II enzyme activity in BMDM with 2-DG treatment under the condition of two cells co-culture (n=4). (G) The lactate and pyruvate levels in BMDM with 2-DG pre-treatment under the condition of two cells co-culture (n=4). (H) Western blotting analysis of iNOS and Mincle in BMDM with 2 hours pre-treatment with glycolysis inhibitor 2-DG (10 mM) (n=6). (I) Real-time PCR analysis of the mRNA expression levels of MCP-1, IL-1β, and TNF-α in BMDM with 2 hours pre-treatment with glycolysis inhibitor 2-DG (10 mM) (n=6). All data above are represented as means ± SD. Compared to Sham or Ctrl group, *P <0.05, **P <0.01, ***P <0.001; Compared to Vehicle group, #*P* <0.05, ##*P* <0.01, ###*P* <0.001.

Growing evidence indicated that the metabolic reprogramming plays a vital role in the progression of renal diseases 21, thus, we hypothesized that macrophage metabolic reprogramming may be involve in the AKI to CKD transition. Interestingly, we found that the renal glycolytic enzymes HK II activity, renal lactate and pyruvate levels were markedly elevated in mice with 35 minutes I/RI (Fig. 5A-5B) and in mice with UUO (Supplementary Fig. 3A-3B), suggesting that glycolysis may be the primary metabolic mode during AKI to CKD transition. Then, the glycolytic enzymes HK II and the glucose transporter GLUT1 proteins were detected. Intriguingly, we found that the expressions of HK II and GLUT1 mRNA were increased in mice with 35 minutes I/RI (Fig. 5C) and in mice with UUO (Supplementary Fig. 3C). Meanwhile, its protein expression was also confirmed by Western blotting (Fig. 5D and supplementary Fig. 3D-3E). Next, the glycolytic enzymes HK II and the glucose transporter GLUT1 of pro-inflammatory macrophage* in vitro* was explored (Fig. 5E). Consistent with* in vivo* experiments, the glycolytic enzymes HK II activity and the lactate and pyruvate levels were significantly increased in the BMDM with co-culture under the long-hypoxia condition (Supplementary Fig. 3F-3G). Further, to study the functional effect of glycolysis on macrophage phenotype, the glycolytic inhibitor 2-DG was used. As expected, it is observed that the macrophage glycolysis (Fig. 5F-5G) and pro-inflammatory macrophage was markedly inhibited (Fig. 5H and supplementary Fig. 3H-3I). More importantly, the expressions of inflammatory factors were also inhibited in BMDM (Fig. 5I). These results suggest that metabolic reprogramming of glycolysis is essential for maintaining pro-inflammatory macrophage phenotype.

### HIF-1 plays a crucial role in macrophage glycolysis during AKI to CKD transition

**Figure 6:**
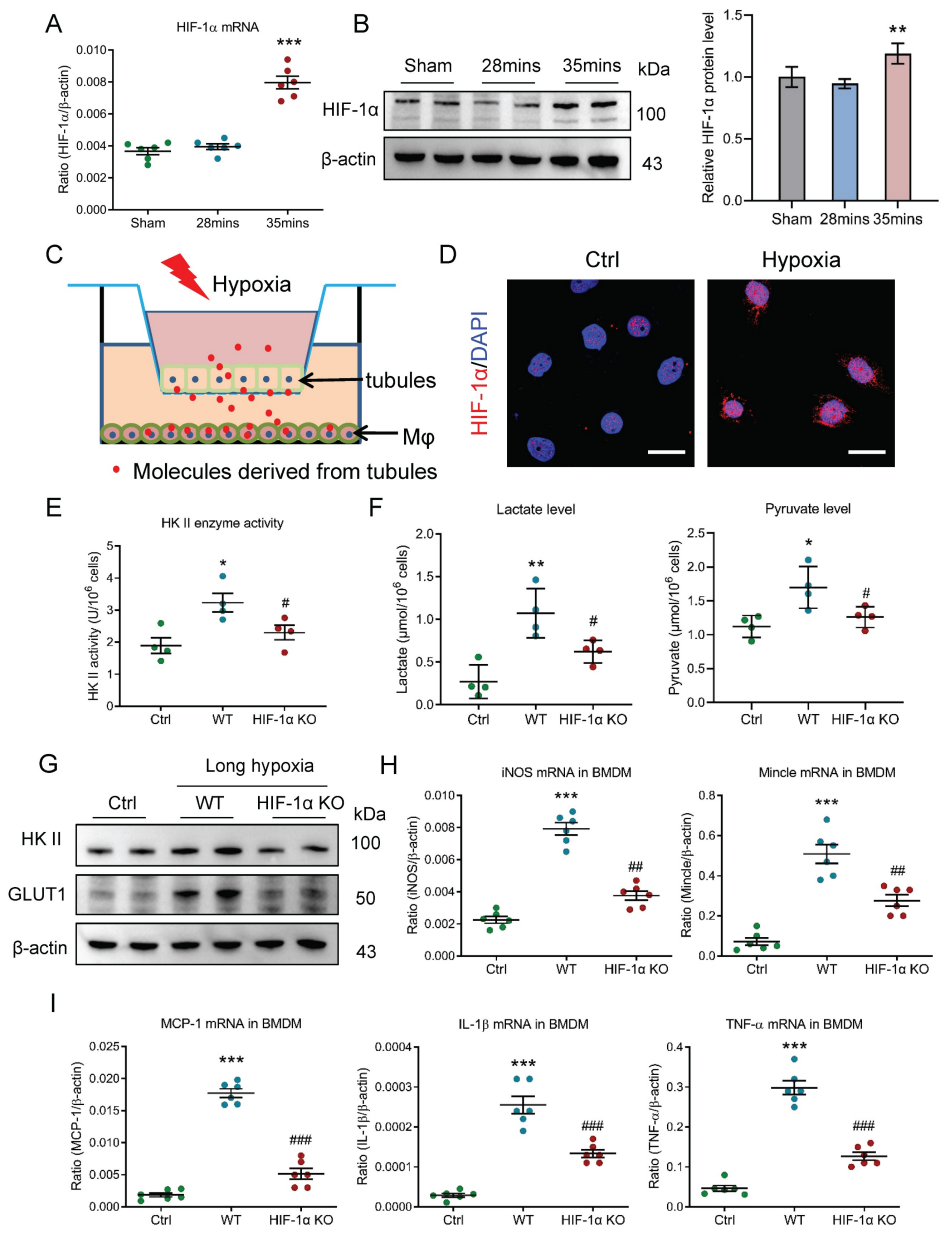
** HIF-1 plays a crucial role in macrophage glycolysis during AKI to CKD transition.** (A) The level of HIF-1α mRNA (n=6). (B) Western blotting analysis and densitometric analysis of HIF-1α in kidney (n=6). (C) Schematic illustration of the mTECs and macrophage co-culture *in vitro* experiment. (D) Representative images of HIF-1α staining of BMDM with co-culture under short or long hypoxia. Scale bars, 20 μm. (E) The HK II enzyme activity in BMDM with *HIF-1α* knockout (n=4). (F) The lactate and pyruvate levels in BMDM with *HIF-1α* knockout (n=4). (G) Western blotting analysis of HK II and GLUT1 in BMDM with *HIF-1α* knockout (n=6). (H) Real-time PCR analysis of the mRNA expression levels of iNOS and Mincle in BMDM with *HIF-1α* knockout (n=6). (I) Real-time PCR analysis of the mRNA expression levels of MCP-1, IL-1β, and TNF-α in BMDM with *HIF-1α* knockout (n=6). All data above are represented as means ± SD. Compared to Sham or Ctrl group, *P <0.05, **P <0.01, ***P <0.001; Compared to WT group, #*P* <0.05, ##*P* <0.01, ###*P* <0.001.

Then, the molecular mechanism of glycolysis metabolic reprogramming in macrophage during AKI to CKD transition was explored. Considering HIF-1, as a main transcriptional regulator of metabolic adaptation to changes in the hypoxia environment, is a pivotal modulator of the metabolic reprogramming, we subsequently explored its effects on macrophage glycolysis under the condition of AKI to CKD transition. We observed that the HIF-1α mRNA expression were increased in mice with 35 minutes I/RI (Fig. 6A) and in mice with UUO (Supplementary Fig. 4A). Meanwhile, its protein expression was also confirmed by Western blotting (Fig. 6B and Supplementary Fig. 4B). Then, the* in vitro* experiments were studied (Fig. 6C) and the HIF-1α expression was increased and was predominantly expressed in the nucleus of BMDM with long-hypoxia treatment (Fig. 6D). Next, the *HIF-1α* knockout BMDM was used to further study. Interestingly, when the HIF-1α was knocked out, the glycolytic enzymes HK II activity (Fig. 6E), the lactate and pyruvate levels (Fig. 6F), the mRNA and protein expressions of glycolytic enzymes HK II and the glucose transporter GLUT1 were markedly inhibited (Supplementary Fig. 4C, Fig. 6G, and Supplementary Fig. 4D), suggesting that the HIF-1α played a crucial role in macrophage glycolysis. Consequently, the pro-inflammatory phenotype was changed (Fig. 6H) and the inflammatory factors mRNA expressions were also attenuated (Fig. 6I). Taken together, HIF-1α is the pivotal modulator of the macrophage metabolic reprogramming during AKI to CKD transition.

### Myeloid *HIF-1α* knockout alleviates tubulointerstitial fibrosis in AKI to CKD transition

**Figure 7:**
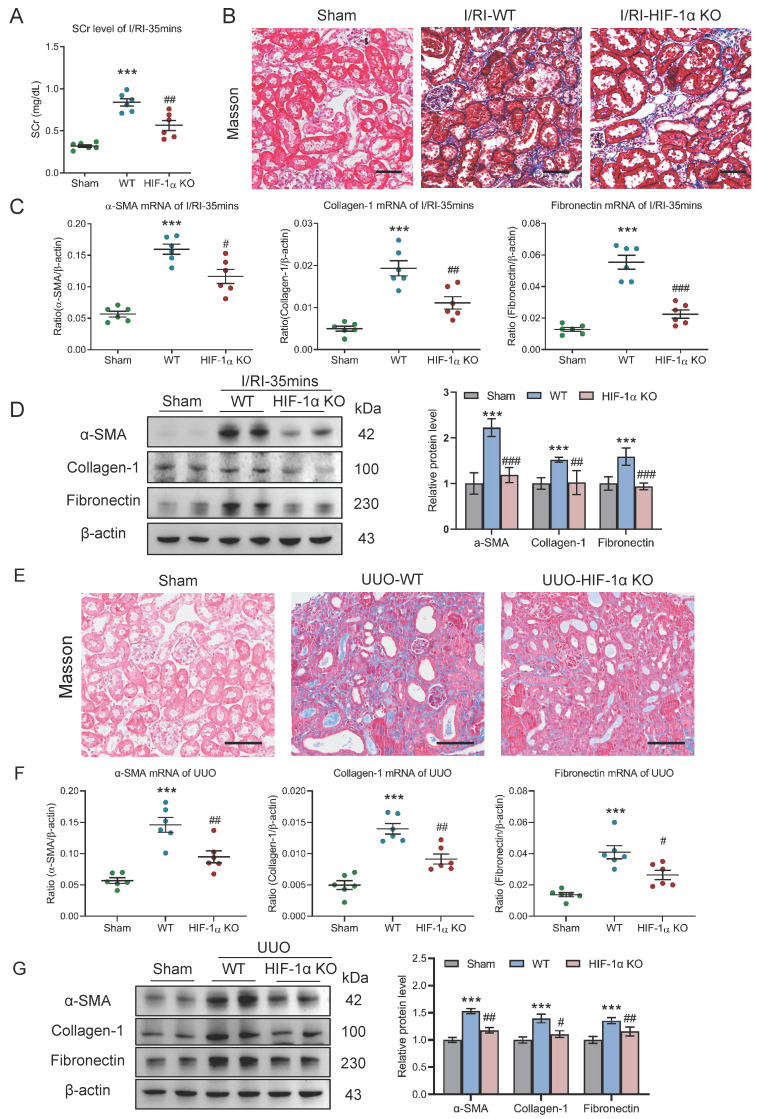
** Myeloid *HIF-1α* knockout alleviates tubulointerstitial fibrosis in AKI to CKD transition.** (A) The SCr levels in myeloid *HIF-1α* knockout mice with I/RI (n=6). (B) Histological changes (Masson's trichrome staining) of kidney from myeloid *HIF-1α* knockout mice with I/RI. Scale bar, 50 μm. (C) Real-time PCR analysis of the mRNA expression levels of α-SMA, collagen-1 and fibronectin in kidney from myeloid *HIF-1α* knockout mice with I/RI (n=6). (D) Western blotting analysis and densitometric analysis of fibrotic markers in kidney from myeloid *HIF-1α* knockout mice with I/RI (n=6). (E) Histological changes (Masson's trichrome staining) of kidney from myeloid *HIF-1α* knockout mice with UUO. Scale bars, 100 μm. (F) Real-time PCR analysis of the mRNA expression levels of α-SMA, collagen-1 and fibronectin in kidney (n=6). (G) Western blotting analysis and densitometric analysis of α-SMA, collagen-1 and fibronectin of kidney from myeloid *HIF-1α* knockout mice with UUO (n=6). All data above are represented as means ± SD. Compared to Sham group, ***P <0.001; Compared to WT group, #*P* <0.05, ##*P* <0.01, ###*P* <0.001.

To clarify the potential effect of the HIF-1α-mediated macrophage glycolysis on the AKI to CKD transition *in vivo*, myeloid *HIF-1α* knockout mice with 35 minutes I/RI was performed. Strikingly, we found that the SCr level was significantly decreased on day 21 after 35 minutes I/RI in the myeloid *HIF-1α* knockout mice (Fig. 7A). Similarly, Masson staining also demonstrated ameliorated fibrotic lesions (Fig. 7B). Moreover, myeloid *HIF-1α* knockout attenuates the increased expression of α-SMA, collagen-1, and fibronectin at the mRNA and protein level in renal cortical tissues, as demonstrated by RT-PCR and Western blotting (Fig. 7C and 7D).

Next, we examined the outcomes of myeloid* HIF-1α* knockout in UUO mice. As expected, ameliorated fibrotic lesions were observed, as evidenced by the Masson staining (Fig. 7E). Concomitantly, there were significant decreases in mRNA and protein expression of renal fibrotic markers in myeloid *HIF-1α* knockout mice with UUO (Fig. 7F-7G). Therefore, these data indicate that HIF-1α-mediated macrophage glycolysis was the exact molecular mechanism of CKD progression after AKI.

### HIF-1 is transcriptionally regulated by NF-κB in pro-inflammatory macrophage

**Figure 8:**
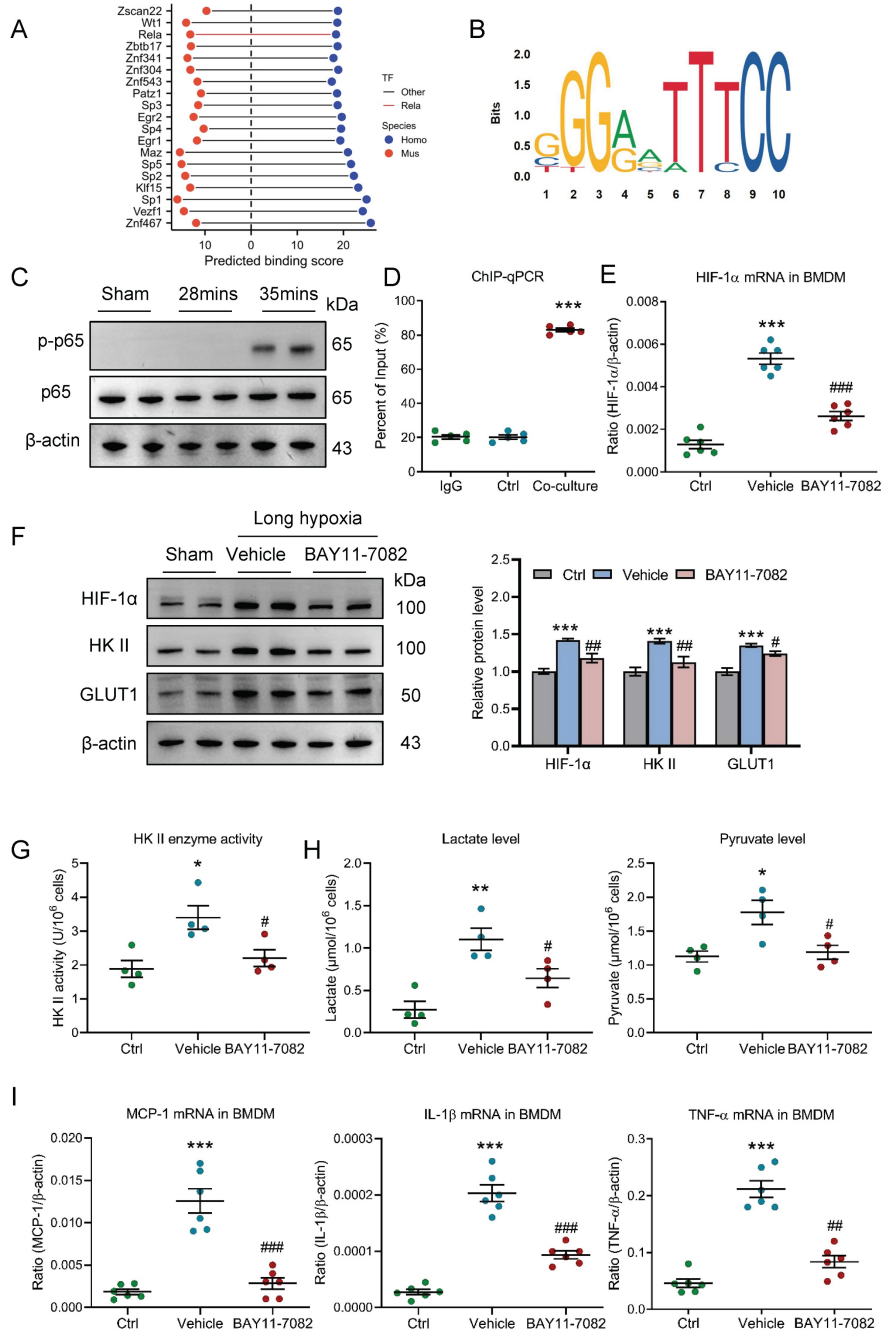
** HIF-1 is transcriptionally regulated by NF-κB in pro-inflammatory macrophage.** (A) Top 20 transcription factors of HIF-1α predicted by TFDB database. (B) Motifs of human HIF-1α binding with p65. (C) Western blotting analysis of phosphorylated-p65 in kidney with I/RI. (D) ChIP-quantitative PCR revealed that p65 bound to the HIF-1α promoter in BMDM with co-culture under short or long hypoxia (n=5). (E) The level of HIF-1α mRNA in BMDM with NF-κB inhibitor BAY11-7082 treatment (n=6). (F) Western blotting analysis of HIF-1α, HK II and GLUT1 in BMDM (n=6). (G) The HK II enzyme activity in BMDM with NF-κB inhibitor BAY11-7082 treatment (n=4). (H) The lactate and pyruvate levels in BMDM with NF-κB inhibitor BAY11-7082 treatment (n=4). (I) Real-time PCR analysis of the mRNA expression levels of MCP-1, IL-1β, and TNF-α in BMDM with NF-κB inhibitor BAY11-7082 treatment (n=6). All data above are represented as means ± SD. Compared to Ctrl group, *P <0.05, **P <0.01, ***P <0.001; Compared to Vehicle group, #*P* <0.05, ##*P* <0.01, ###*P* <0.001.

Next, the exact mechanism that regulates HIF-1 in pro-inflammatory macrophage was further investigated under conditions of AKI to CKD transition. Given that pro-inflammatory macrophage is unable to utilize oxygen, HIF-1*α* may be regulated at the transcriptional level. We collected a list of predicted transcription factors (TFs) that could directly bind to the promoter region of HIF-1 from animal TFDB 4.0 database (https://guolab.wchscu.cn/AnimalTFDB4/) (Table S1). After reserving highest-affinity TF per binding site, and computing the intersection, we identified over 200 evolutionarily conserved TFs shared between human and mouse. Then, the top 20 TFs were ranked by cumulative binding scores across both species, revealing NF-κB (Rela/p65) as a top-ranked regulator (Fig. 8A). Two promoter sites of human HIF-1α binding with p65 at 85% confidence were searched from Jaspar database (https://jaspar.elixir.no/) (Fig. 8B). Thus, we speculated that NF-κB may play a vital role in regulating HIF-1 in macrophage. Interestingly, we found that the p-p65 expression was significantly increased in kidney with 35 minutes I/RI (Fig. 8C and supplementary Fig. 5A) and in kidney with UUO (Supplementary Fig. 5B). Further, as shown in figure 8D, we found that NF-κB p65 could also directly bind to the HIF-1α promoter in BMDM with co-culture under long hypoxia. More interestingly, the HIF-1α mRNA and protein levels were markedly decreased in BMDM with NF-κB specific inhibitor BAY11-7082 administration (Fig. 8E and 8F). Then, the functional effect of NF-κB pathway on HIF-1α-mediated pro-inflammatory phenotype and metabolic reprogramming was investigated. As expected, when the NF-κB pathway was inhibited using BAY11-7082, the glycolytic enzymes HK II activity (Fig. 8G), the lactate and pyruvate levels (Fig. 8H), the glycolytic enzymes HK II and the glucose transporter GLUT1 was decreased in the BMDM (Fig. 8F and supplementary Fig. 5C). Similarly, the pro-inflammatory phenotype markers and inflammation factor mRNA expressions were also decreased (Supplementary Fig. 5D and Fig. 8I). Collectively, HIF-1-medaited metabolic reprogramming is regulated by NF-κB in pro-inflammatory macrophage.

### Blockage of NF-κB ameliorates tubulointerstitial fibrosis in AKI to CKD transition

**Figure 9:**
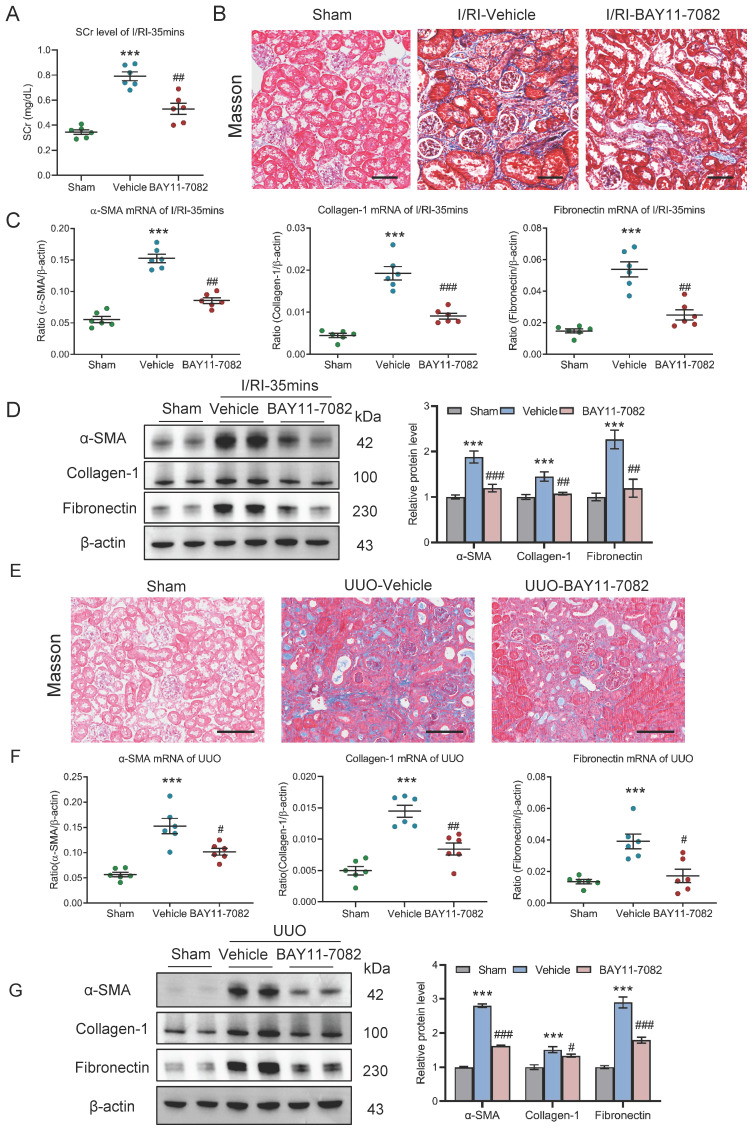
** Blockage NF-κB ameliorates tubulointerstitial fibrosis in AKI to CKD transition.** (A) The SCr levels in mice with I/RI (n=6). (B) Histological changes (Masson's trichrome staining) of kidney from I/RI mice with NF-κB inhibitor BAY11-7082 treatment. Scale bar, 50 μm. (C) Real-time PCR analysis of the mRNA expression levels of α-SMA, collagen-1 and fibronectin in kidney from I/RI mice NF-κB inhibitor BAY11-7082 treatment (n=6). (D) Western blotting analysis and densitometric analysis (n=6). (E) Histological changes (Masson's trichrome staining) of kidney from UUO mice with BAY11-7082 treatment. Scale bars, 100 μm. (F) Real-time PCR analysis of the mRNA expression levels of α-SMA, collagen-1 and fibronectin in kidney (n=6). (G) Western blotting analysis and densitometric analysis of α-SMA, collagen-1 and fibronectin of kidney from UUO mice with BAY11-7082 treatment (n=6). All data above are represented as means ± SD. Compared to Sham group, ***P <0.001; Compared to Vehicle group, #*P* <0.05, ##*P* <0.01, ###*P* <0.001.

Finally, the effect of NF-κB pathway on AKI to CKD transition was explored *in vivo*. NF-κB pathway inhibition in mice with 35 minutes I/RI was performed by intraperitoneal injections of NF-κB inhibitor BAY11-7082. Firstly, we found that the SCr level was significantly decreased on day 21 after 35 minutes I/RI in the BAY11-7082 mice (Fig. 9A). Similarly, Masson staining also demonstrated ameliorated fibrotic lesions (Fig. 9B). Moreover, BAY11-7082 treatment attenuates the increased expression of α-SMA, collagen-1, and fibronectin at the mRNA and protein level in renal cortical tissues, as demonstrated by RT-PCR and Western blotting (Fig. 9C and 9D).

Moreover, the outcomes of NF-κB inhibition in UUO-induced AKI to CKD transition model were explored. Interestingly, we found that fibrotic lesions were significantly ameliorated (Fig. 9E). Meanwhile, increased expression of α-SMA, collagen-1, and fibronectin at the mRNA and protein level in renal cortical tissues were markedly reversed (Fig. 9F-9G). Collectively, these results demonstrate that blockage NF-κB ameliorates tubulointerstitial fibrosis and CKD progression after AKI.

## Discussion

Renal maladaptive repair is a one of the most important characteristic features of AKI to CKD transition. Macrophages are a heterogeneous population of immune cells playing diverse functions, which play a crucial role in renal maladaptive repair. However, the underlying mechanism of orchestrating characterization of macrophage in renal maladaptive repair remains largely unclear. Accordingly, we demonstrated in the present study that pro-inflammatory macrophage is the primary contributor to the renal maladaptive repair in AKI to CKD transition. Mechanistically, HIF-1α, which is transcriptionally regulated by NF-κB, appears to be essential for maintaining pro-inflammatory macrophage phenotype via mediating glycolysis metabolism reprogramming. More generally, our studies provide a novel paradigm in which pro-inflammatory macrophage orchestrates renal maladaptive repair, contributing to the AKI to CKD transition.

In earlier studies, cell-cell crosstalk has been identified to be an important feature for orchestrating the progression of kidney disease 8, 22-23. Although a unique and complex niche including intrinsic renal cells, kidney resident and infiltrated inflammatory cells, extracellular matrix, extracellular vesicles, soluble factors and metabolites, is formed during AKI to CKD transition, increasing evidence suggests that TEC with maladaptive repair is considered as a key instigator of AKI to CKD transition 24. For instance, Doke *et al.* 25 identified a subset of TECs with a profibrotic-inflammatory phenotype characterized by recruiting basophils as main contributors to the development of renal fibrosis using single-cell analysis. More importantly, recently, Melchinger *et al.* 26 demonstrated that during AKI to CKD transition, *VCAM-1+* proximal tubule (also called failed tubule recovery) interacted with immune cell to promote further renal injury. In addition, our previously study also demonstrated that exosome mediates the cross-talk between TECs and macrophages in tubulointerstitial inflammation 14. Thus, in the present, using the classical I/RI-induced AKI to CKD transition *in vivo* model and a novel tubule-macrophage co-culture* in vitro* model, we indicated that maladaptive TEC-macrophage interaction was a key instigator of AKI to CKD transition. The TECs-macrophage cross-talk pattern may be the basis for their important pathophysiological roles in AKI to CKD transition.

It has been recognized that macrophages manifest a high degree of heterogeneity, exhibiting distinct phenotypic and functional traits in response to diverse stimuli within the local microenvironment 27. Indeed, the roles of macrophage subpopulation heterogeneity in kidney inflammation and fibrosis have been preliminary explored. For instance, arginase-1-positive macrophages were found to promote tubular proliferation and renal repair following early injury 28. Our group also identified a novel extracellular matrix remodeling macrophage subset that initiated and amplified renal fibrosis in CKD progression following AKI 18. However, the underlying mechanism of orchestrating characterization of macrophage in AKI to CKD transition has not been previously described. Given the dynamic and complex microenvironment during the AKI to CKD transition, macrophages may also exhibit unique phenotype. Strikingly, we found that macrophages with pro-inflammatory phenotype are involved in AKI to CKD transition. More importantly, we indicated that depletion of macrophages with clodronate liposomes attenuates renal fibrosis on AKI-CKD transition, suggesting that pro-inflammatory macrophage play a crucial role in the AKI to CKD transition. It was consistent with our previous findings that pro-inflammatory Mincle receptor in macrophage contributes to the unresolved inflammation during the transition from AKI to CKD 29. Therefore, pro-inflammatory macrophage infiltration may be an inherent characteristic of CKD progression following AKI, deepening our understand on the pathophysiological changes. To our knowledge, this study is the first to investigate the underlying mechanism of orchestrating characterization of macrophage. However, the precise regulatory pathway for pro-inflammatory macrophage has not been described in the setting of AKI to CKD transition.

Previously, compelling evidence has suggested that macrophage phenotypes and functions are intimately linked to metabolic reprogramming. For instance, by analyzing single-cell RNA sequencing data of renal myeloid cells, an increased gene expression in glycolytic pathway in myeloid cells that are critical for renal inflammation and fibrosis was found 30. More interestingly, macrophage glycolysis metabolic reprogramming presents a therapeutic target in autoimmune kidney diseases 31-32. Here, we first discovered that glycolysis is essential for maintaining pro-inflammatory macrophage phenotype during AKI to CKD transition. Further, we demonstrated that HIF-1 was the crucial contributor to regulating macrophage glycolysis, which was consistent with some of previous findings reported by Jia et al 33. Meanwhile, relevant to our findings, Liu *et al.* 34 indicated that proinflammatory signal shifts macrophage metabolism from Myc-dependent to HIF-1α-dependent. Therefore, HIF-1α-mediated glycolysis metabolic reprogramming may represent a novel mechanism for regulating macrophage phenotype during AKI to CKD transition. In addition, we also found that myeloid *HIF-1α* knockout alleviates AKI to CKD transition. To our knowledge, this study has demonstrated for the first time that myeloid HIF-1α is a critical regulator for AKI to CKD transition.

Then, the molecular mechanism of regulating macrophage HIF-1 pathway was explored. Traditionally, HIF-1 is regulated by an oxygen-dependent degrading pathway. However, compelling evidence demonstrated that under normoxic conditions, stable expression of HIF-1 in macrophages was found 35-36, thus, the macrophage HIF-1 pathway may be regulated by an oxygen-independent pathway. Indeed, the HIF expression could be modulated by noncoding RNAs 37-38. NF-κB, a characteristic proinflammatory transcription factor, can bind to specific sequences in the promoter or enhancer regions of target genes. Increasing evidence has indicated that NF-κB is a critical transcriptional activator of HIF-1α 39. However, it is still unknown whether HIF-1α was transcriptionally regulated by the NF-κB in macrophage during AKI to CKD transition. In the present study, we demonstrated that the induction of HIF-1α required NF-κB activity and that HIF-1α was a direct transcriptional target of NF-κB in pro-inflammatory macrophage. Thus, our findings enrich the regulatory mechanisms of the transcriptional factor HIF pathway. Collectively, these results signify a novel NF-κB-HIF-1 signaling pathway in pro-inflammatory macrophage during AKI to CKD transition.

So, what is the mechanism through which pro-inflammatory macrophages promote renal maladaptive repair during the progression of AKI to CKD. As an innate immune cell, in addition to directly secreting inflammatory mediators to promote renal inflammation and fibrosis, macrophages could also function through an immune-independent manners. Recently, Peng *et al.* 40 demonstrated that macrophage promotes fibroblast activation and kidney fibrosis by assembling a vitronectin-enriched microenvironment in fibrotic kidney after unilateral I/RI or UUO. Moreover, it was also found that macrophage has phagocytic functions in kidney tissue 41. Of note, given the complex functions of macrophages, they may also exert their effects on AKI to CKD transition through other unknown pathways, such as releasing exosome 42-43. Thus, the mechanism through which pro-inflammatory macrophages promote the progression of AKI to CKD needs further investigation.

To date, no effective therapy has been developed to alter the natural history of AKI to CKD transition, which poses significant clinical challenges for clinicians. Exploring kidney repair approach may be one of the most promising therapeutic strategies 44-45. Emerging evidence indicates that macrophage phenotype plays a central role in kidney repair and determines the outcomes of kidney diseases. For instance, in renal fibrosis, M2a-type macrophage promotes disease progression, while M2c-type macrophage possesses potent anti-inflammatory functions and promotes tissue repair 46. More interestingly, precisely regulating M2 subtype macrophages is an effective therapeutic approach for renal fibrosis resolution 47. Impressively, in our study, we demonstrated that blockage NF-κB-HIF-1 pathway-mediated pro-inflammatory macrophage did protect against the tubulointerstitial fibrosis, which significantly ameliorates AKI to CKD transition. Relevant to our findings, Chen *et al.* 48 indicated that pharmacological targeting macrophage pro-inflammatory activity and proliferation attenuate kidney fibrosis in the UUO mice. Therefore, our results indicated that macrophages phenotype serves as a novel therapeutic target for delaying the AKI to CKD transition.

Although macrophage phenotype has been recognized as a critical determinant of the adaptive or maladaptive responses following AKI, modification of macrophages phenotype is still challenging in clinical practice, particularly under a dynamic microenvironment. Thus, understanding of the phenotypic plasticity of macrophages across diverse physiological conditions is a prerequisite for advancing precision medicine. Moreover, identification of targeted pharmacological agents capable of selectively engaging macrophages to enhance adaptive repair of kidney is warrant. Here, we systemically described the underlying mechanism of orchestrating characterization of macrophage in renal maladaptive repair, which provide important evidence for the targeted regulation of macrophage phenotype, potentially advancing immunotherapy strategies for kidney diseases.

**Figure 10:**
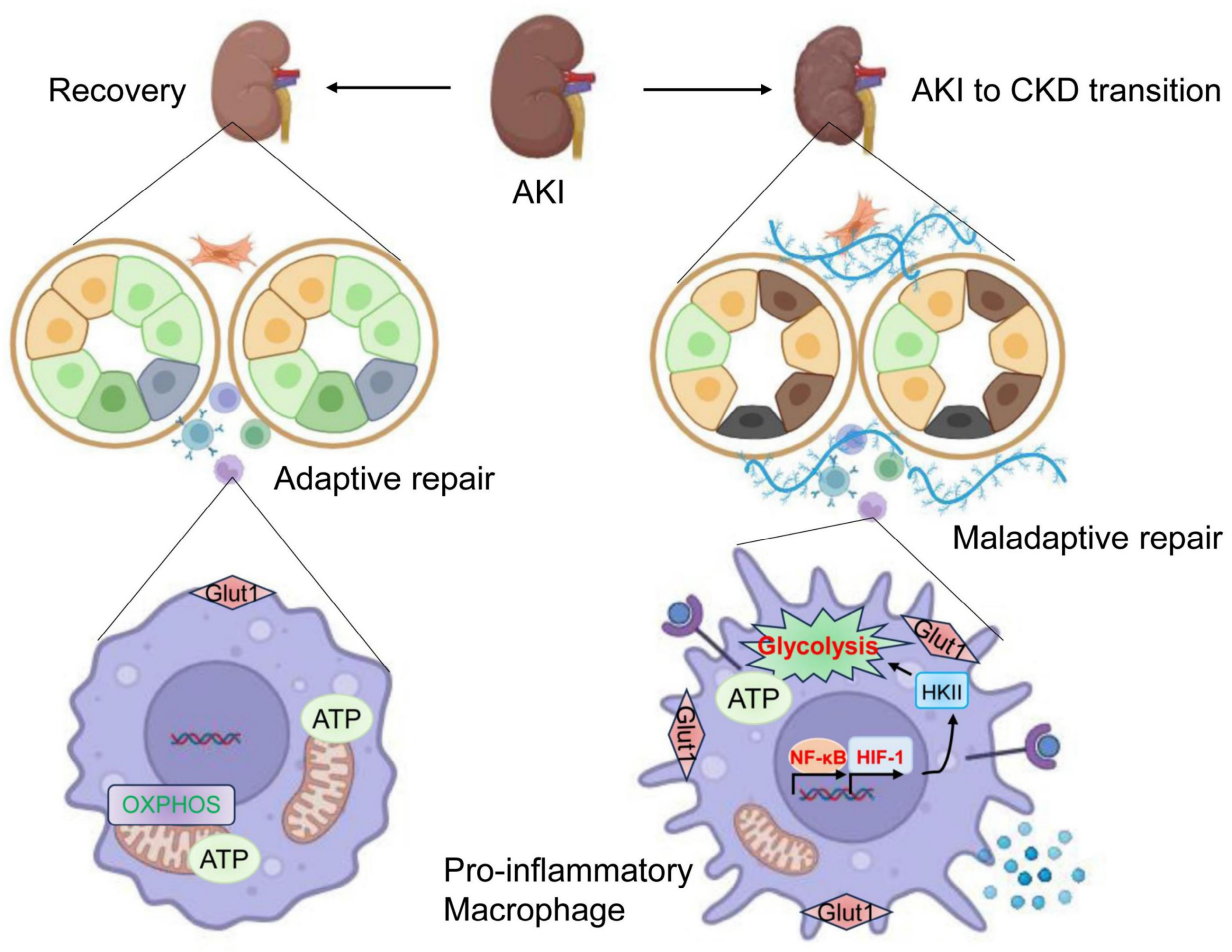
** Schematic illustration of the exact mechanism of orchestrating characterization of macrophage in renal maladaptive repair.** During the AKI to CKD transition, pro-inflammatory macrophage is the primary contributor to the renal maladaptive repair. Mechanistically, HIF-1α, which is transcriptionally regulated by NF-κB, appears to be essential for maintaining pro-inflammatory macrophage phenotype via mediating glycolysis metabolism reprogramming. Then, the release of increased inflammatory cytokines results in tubulointerstitial fibrosis and AKI to CKD transition. Image was created with BioRender.com. with permission.

In conclusion, in the present study, the underlying mechanism of orchestrating characterization of macrophage in renal maladaptive repair following AKI was studied (Figure 10). Accordingly, we discovered that pro-inflammatory macrophage is the primary contributor to the renal maladaptive repair in AKI to CKD transition. Mechanistically, HIF-1α, which is transcriptionally regulated by NF-κB, appears to be essential for maintaining pro-inflammatory macrophage phenotype via mediating glycolysis metabolism reprogramming. More generally, our studies provide a novel paradigm in which pro-inflammatory macrophage orchestrates renal maladaptive repair, contributing to the AKI to CKD transition. These findings provide unique insights into interdigitating mechanisms of renal maladaptive repair in AKI to CKD transition. Interrupting the NF-κB-HIF-1 signaling-mediated pro-inflammation macrophage represents a promising therapeutic target for AKI to CKD transition.

## Supplementary Material

Supplementary figures.

Supplementary table 1.

## Figures and Tables

**Table 1:** Primers used for quantitative RT-PCR

Primer	Forward (5'-3')	Reverse (5'-3')
Ms β-actin	GAGACCTTCAACACCCCAGC	ATGTCACGCACGATTTCCC
Ms IL-1β	TGCCACCTTTTGACAGTGATG	AAGGTCCACGGGAAAGACAC
Ms TNF-α	CATCTTCTCAAAATTCGAGTGACAA	TGGGAGTAGACAAGGTACAACCC
Ms MCP-1	CTTCTGGGCCTGCTGTTCA	CCAGCCTACTCATTGGGATCA
Ms Mincle	ACCAAATCGCCTGCATCC	CACTTGGGAGTTTTTGAAGCATC
Ms iNOS	CAGATCGAGCCCTGGAAGAC	CTGGTCCATGCAGACAACCT
Ms HK II	TGATCGCCTGCTTATTCACGG	AACCGCCTAGAAATCTCCAGA
Ms GLUT1	GGTGTGCAGCAGCCTGTGTA	CCATCGGCTCCGGTATCGTCAACAC
Ms HIF-1α	AGATTCTGTTTGTTGAAGGGAG	AGGTGGATATGTCTGGGTTGA
Ms α-SMA	CAGCAAACAGGAATACGACGAA	AACCACGAGTAACAAATCAAAGC
Ms collagen-1	GTCAGACCTGTGTGTTCCCTACTCA	TCTCTCCAAACCAGACGTGCTTC
Ms Fibronectin	GCAAGAAGGACAACCGAGGAAA	GGACATCAGTGAAGGAGCCAGA

GLUT1, glucose transporter 1; HIF-1α, hypoxia-inducible factor-1α; IL-1β, interleukin-1β; MCP-1, monocyte chemoattractant protein-1; TNF-α, tumor necrosis factor-α; iNOS, inducible nitric oxide synthase; HK II, hexokinase II; α-SMA, α-smooth muscle actin.
