# Supplementary material for: HIF-1-mediated macrophage metabolic reprogramming promotes AKI to CKD transition
Source: Int J Biol Sci. 2025 Sep 22;21(13):5936–55. doi: 10.7150/ijbs.111238 (PMC12509917; doi:10.7150/ijbs.111238)
Supplement: Supplementary file 1 — Supplementary figures. [file ijbsv21p5936s1.pdf]

Supplementary Figure 1

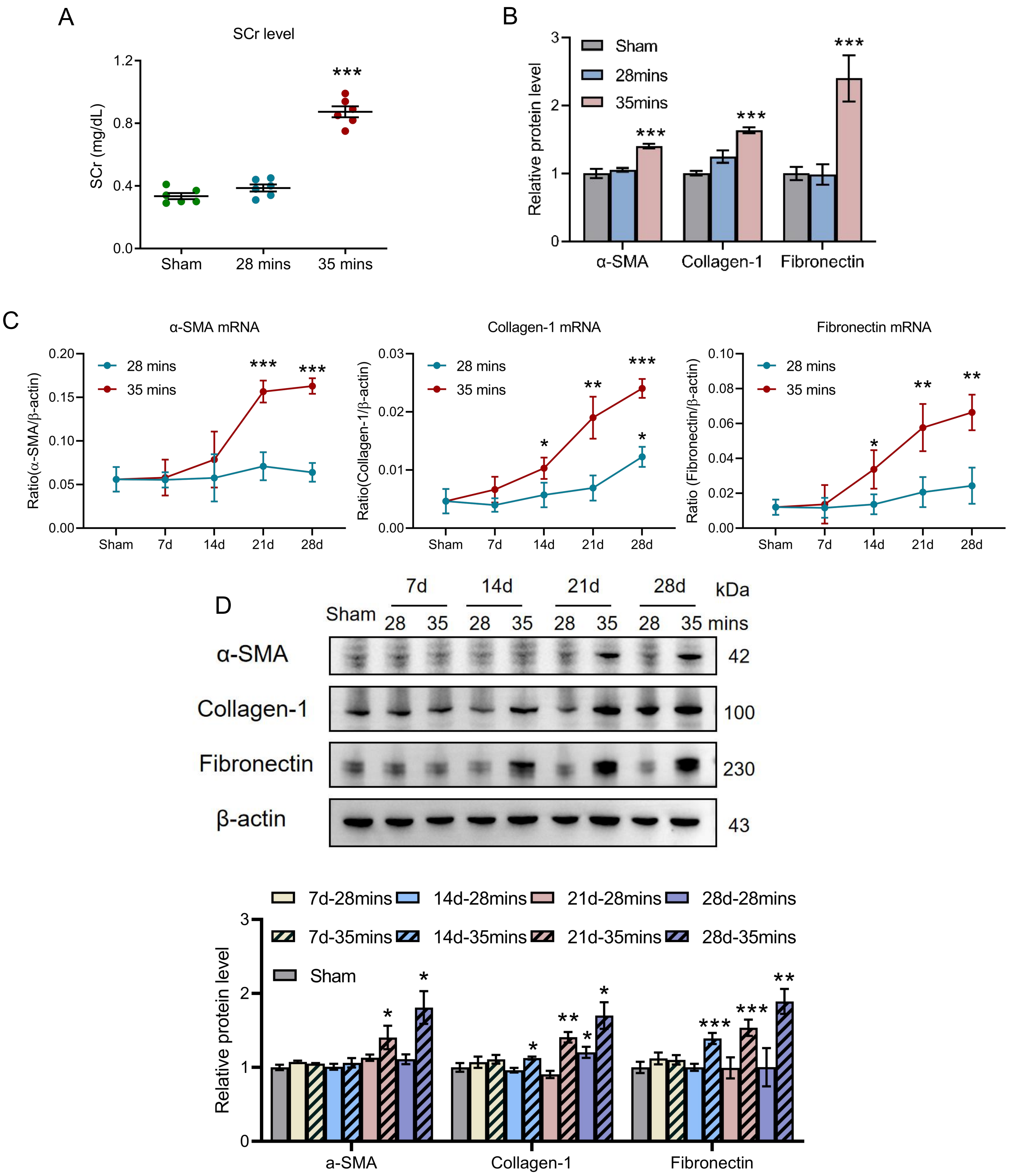

Supplementary Figure 2

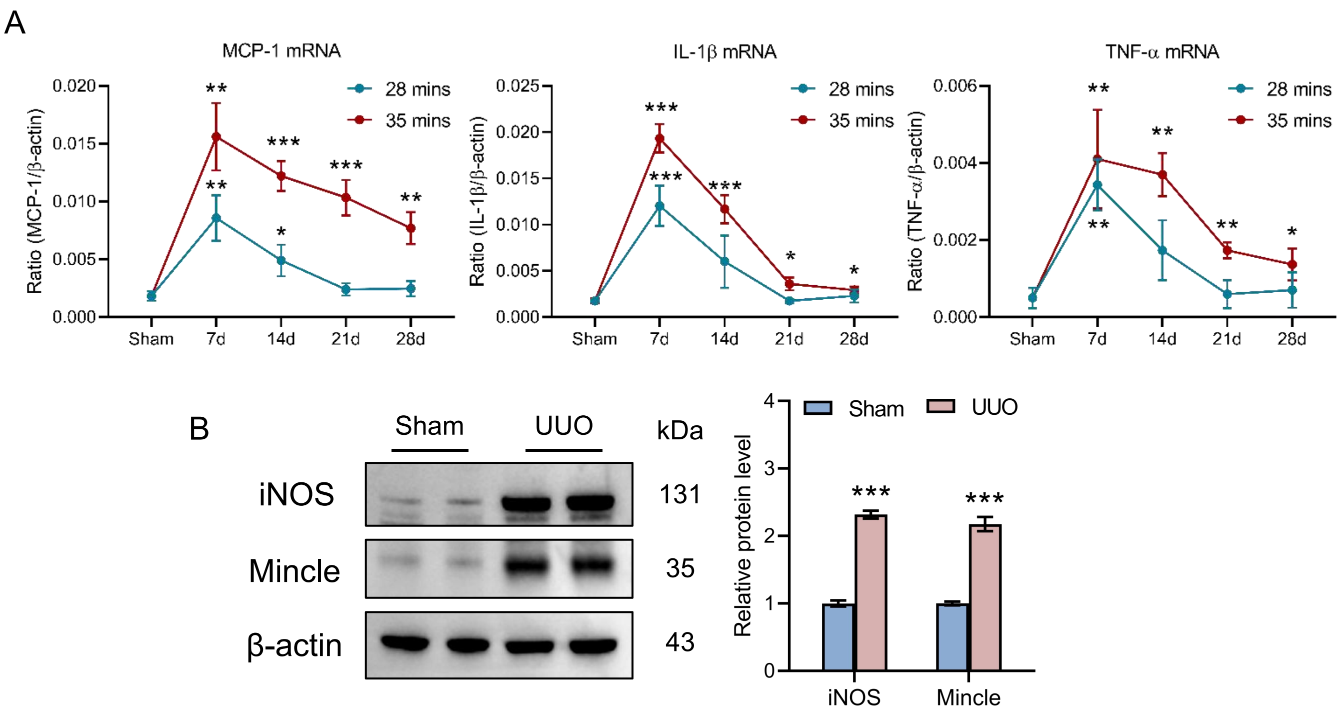

Supplementary Figure 3

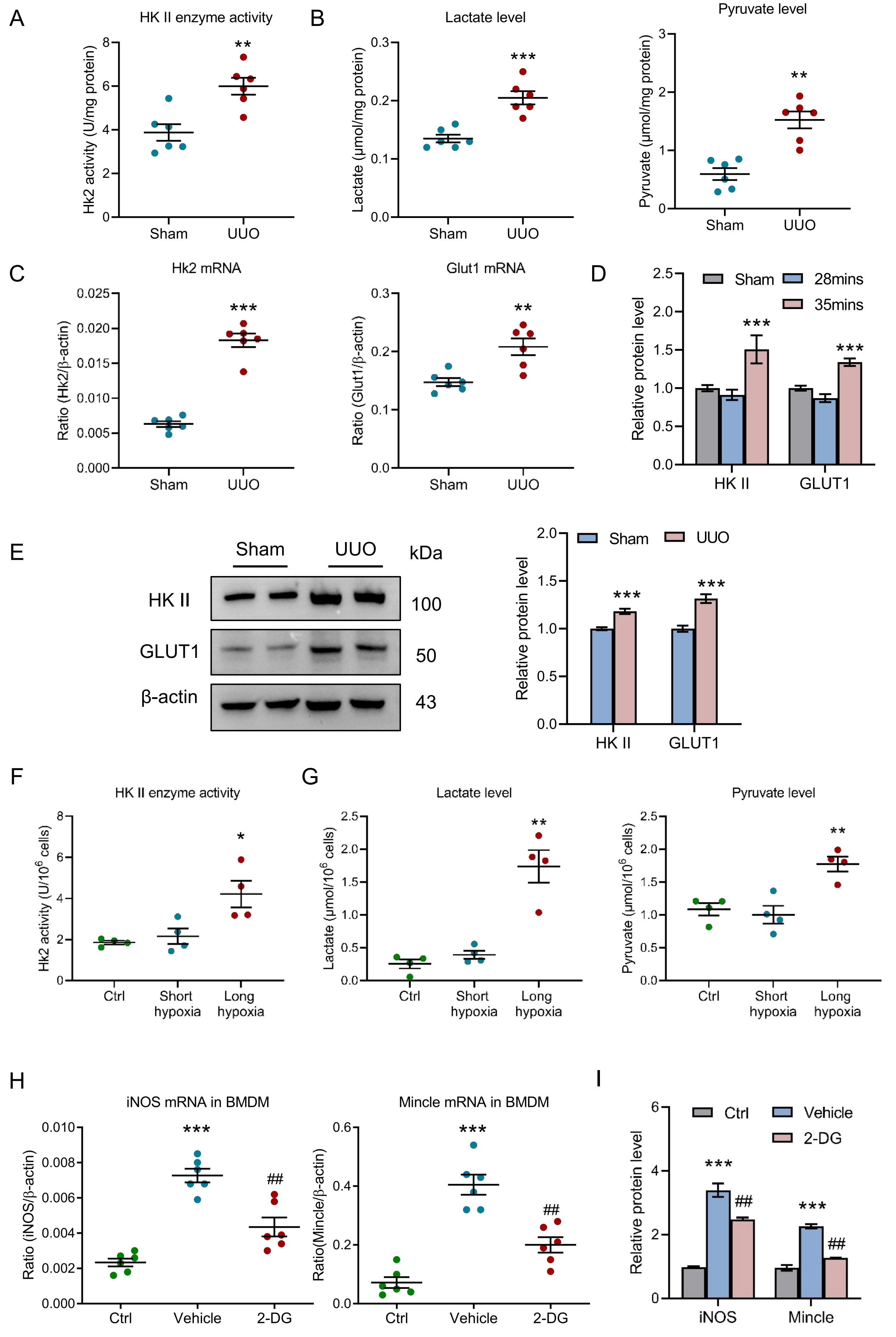

Supplementary Figure 4

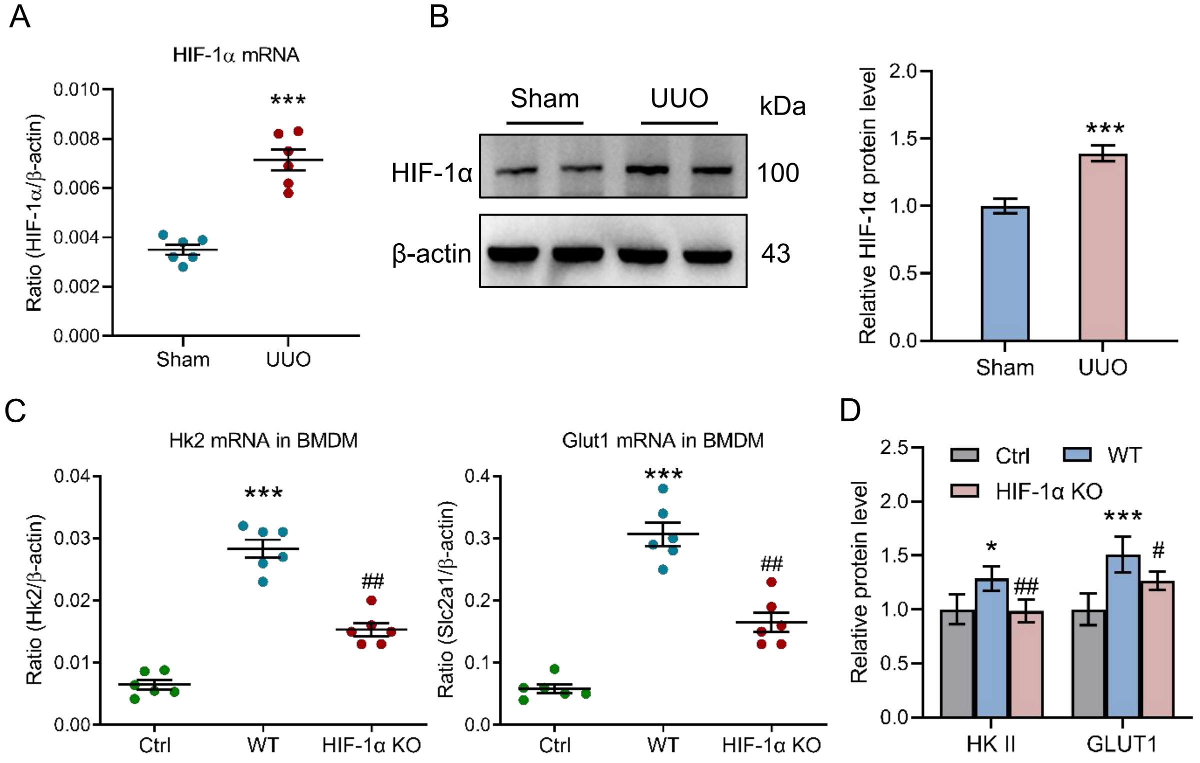

Supplementary Figure 5

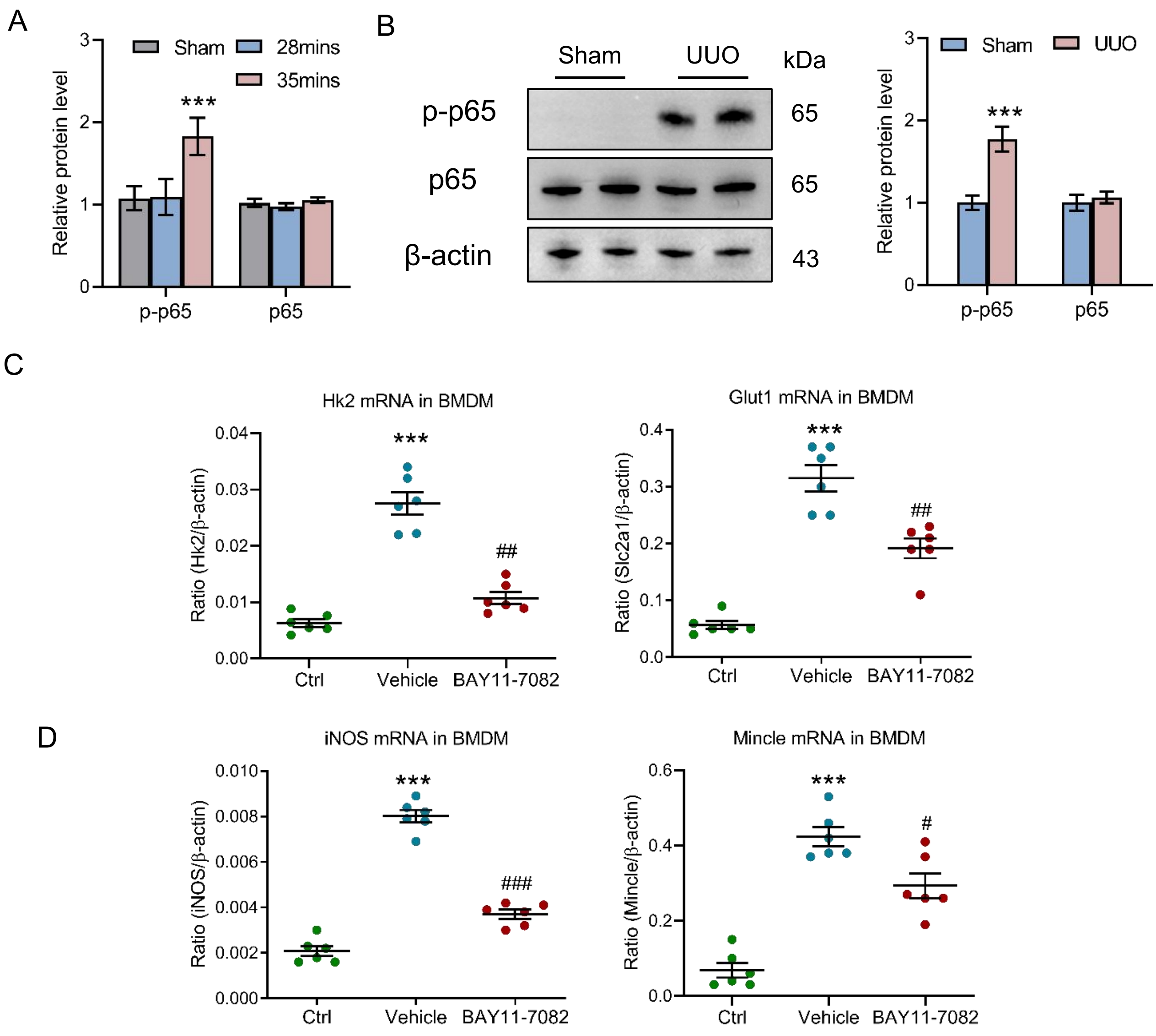

Supplementary figure 1. Renal maladaptive repair following AKI is found in AKI to CKD transition model. (A) Elevated serum creatinine was found in mice with 35 minutes I/RI on day 21 (n=6). (B) The densitometric analysis of Western blotting shows that significant renal fibrosis was observed in kidneys from mice with 35 minutes I/RI (n=6). (C) A longitudinal analysis of fibrosis markers mRNA at multiple time points post-I/RI were showed (n=5). (D) A longitudinal analysis of fibrosis markers protein at multiple time points post-I/RI were showed (n=5). All data above are represented as means ± SD. Compared to Sham group, **P* <0.05, ***P* <0.01, ****P* <0.001.

Supplementary figure 2. Persistent inflammation and pro-inflammatory macrophage phenotype are observed in AKI to CKD transition. (A) A longitudinal analysis of inflammatory cytokines mRNA at multiple time points post-I/RI were showed (n=5). (D) Western blot analysis of iNOS and Mincle protein in the UUO-induced AKI to CKD transition model (n=6). All data above are represented as means ± SD. Compared to Sham group, **P* <0.05, ***P* <0.01, ****P* <0.001.

Supplementary figure 3. Glycolysis is crucial for maintaining pro-inflammatory macrophage phenotype. (A) The HK II enzyme activity in kidney with UUO (n=6). (B) The lactate and pyruvate levels in kidney with UUO (n=6). (C) Real-time PCR analysis of the mRNA expression level of HK II and GLUT1 in kidney from mice with UUO (n=6). (D) Densitometric analysis of HK II and GLUT1 in kidney with I/RI (n=6). (E) Western blotting analysis and densitometric analysis of HK II and GLUT1 in kidney with UUO (n=6). (F) The HK II enzyme activity in BMDM with hypoxia treatment (n=4). (G) The lactate and pyruvate levels in BMDM with hypoxia treatment (n=4). (H) Real-time PCR analysis of the mRNA expression levels of iNOS and Mincle in BMDM with 2 hours pre-treatment with glycolysis inhibitor 2-DG (10 mM) (n=6). (I) Densitometric analysis of iNOS and Mincle in BMDM with 2 hours pre-treatment with glycolysis inhibitor 2-DG (10 mM) (n=6). All data above are

represented as means ± SD. Compared to Sham or Ctrl group, **P* <0.05, ***P* <0.01, ****P* <0.001; Compared to Vehicle group, ##*P* <0.01.

Supplementary figure 4. HIF-1 played a crucial role in macrophage glycolysis. (A) The level of HIF-1α mRNA in kidney with UUO (n=6). (B) Western blotting analysis and densitometric analysis of HIF-1α in kidney with UUO (n=6). (C) Real-time PCR analysis of the mRNA expression levels of HK II and GLUT1 in BMDM with HIF-1α knockout (n=6). (D) The densitometric analysis of Western blot shows that glycolysis metabolic reprogramming was inhibited in HIF-1α KO BMDM. All data above are represented as means ± SD. Compared to Sham or Ctrl group **P* <0.05, ****P* <0.001; Compared to WT group, #*P* <0.05, ##*P* <0.01.

Supplementary figure 5. NF-κB is involved in glycolysis of pro-inflammatory macrophage. (A) The densitometric analysis of phosphorylated-p65 (p-p65) expression in kidneys from mice with I/RI (n=6). (B) Western blotting analysis and densitometric analysis of p-p65 in kidney with UUO (n=6). (C) Real-time PCR analysis of the mRNA expression levels of HK II and GLUT1 in BMDM with NF-κB inhibitor BAY11-7082 treatment (n=6). (D) Real-time PCR analysis of the mRNA expression levels of iNOS and Mincle in BMDM with NF-κB inhibitor BAY11-7082 treatment (n=6). All data above are represented as means ± SD. Compared to Sham, or Ctrl ****P* <0.001; Compared to Vehicle group, #*P* <0.05, ##*P* <0.01, ###*P* <0.001.
